# SERCAP: is the perfect the enemy of the good?

**DOI:** 10.1186/s12936-021-03821-z

**Published:** 2021-06-24

**Authors:** Nicholas J. White, François H. Nosten

**Affiliations:** 1 Mahidol Oxford Tropical Medicine Research Unit (MORU), Faculty of Tropical Medicine, Mahidol University, Bangkok, Thailand; 2 Centre for Tropical Medicine and Global Health, Nuffield Department of Medicine, University of Oxford, Oxford, OX3 7BN UK; 3 Shoklo Malaria Research Unit, MORU, Faculty of Tropical Medicine, Mahidol University, Mae Sot, Tak Thailand

## Abstract

Single Encounter Radical Cure and Prophylaxis (SERCAP) describes an ideal anti-malarial drug that cures all malaria in a single dose. This target product profile has dominated anti-malarial drug discovery and development over the past decade. The operational advantage of a single encounter has to be balanced against the need for a high dose, reliable absorption, little variability in pharmacokinetic properties, slow elimination (to ensure curative drug exposures in all patients) and a very low rate of vomiting. The demanding aspirational target may have hindered anti-malarial drug development. Aiming for three-day regimens, as in current anti-malarial treatments, would be better.

Between 2008 and 2011 the Bill and Melinda Gates Foundation (BMGF) sponsored the Malaria Eradication Research Agenda (malERA) initiative. This was described as a “rigorous scientific consultative process to identify knowledge gaps and new tools needed to eradicate malaria globally”. From this process came an ideal. “The ideal malaria eradication drug is a co-formulated drug combination suitable for mass administration that can be administered in a single encounter at infrequent intervals and that results in radical cure of all life cycle stages of all five malaria species infecting humans” [1]. This ideal is termed “Single Encounter Radical Cure and Prophylaxis” or SERCAP. Given the provenance of the consultation, SERCAP has been very influential in guiding anti-malarial drug development over the past decade. It formed the first target product profile (TPP1) for the Medicines for Malaria Venture (MMV: 2017–2021) of which BMGF is the major funder. “Such a single-dose treatment would be effective against resistant strains of malaria, cure clinical malaria, stop transmission and prevent relapses. It would also simplify case management and improve compliance” [2, 3]. No-one disagrees that such a drug or drug combination, were it to be found, would be wonderful. The question we pose is whether setting such a demanding aspirational target has helped or hindered anti-malarial drug development.

Malaria is currently treated with three-day regimens [4]. The various artemisinin combinations in use are generally highly efficacious and well tolerated. Adherence is not perfect but, where measured, it has been satisfactory. Chloroquine treatment is also given over three days, but the 25 mg base/kg total dose can be squeezed into 36 h. The only times malaria has had single dose treatments were when sulfadoxine-pyrimethamine [SP] (or sulfalene-pyrimethamine) and, briefly, mefloquine were used for the treatment of falciparum malaria, but resistance to both emerged rapidly. Single dose treatment sounds good, and has substantial operational advantages, but is it wise for a potentially lethal infection? Three-day regimens have a very valuable inbuilt safety feature. If the first dose is vomited or poorly absorbed, subsequent doses will compensate. This is important as vomiting is common in young children with acute malaria, who bear most of lethal global burden of malaria. Vomiting or regurgitation of medicines is more likely in children with higher parasite burdens who are more ill [4–7]. It is these patients with high parasite burdens, low anti-malarial drug levels, and little background immunity to control their infections, who are the probable source of de-novo anti-malarial drug resistance [8]. To prevent the emergence of anti-malarial drug resistance in falciparum malaria it is particularly important that hyperparasitaemic patients receive a curative drug regimen. We were fortunate with SP, which was very well tolerated and well absorbed, but many anti-malarial drugs do cause nausea, particularly at the high doses that might be needed to ensure a single encounter cure, and resources to observe young children for an hour after anti-malarial drug administration are often unavailable. Three-day anti-malarial drug regimens work very well. The problem with 3-day treatments is not poor adherence, it is poor access, although substantial improvements have been made in recent years.

Providing radical cure for vivax and ovale malaria adds an additional and difficult challenge. The only drugs providing radical cure are the 8-aminoquinolines, but these compounds cause haemolysis in glucose-6-phosphate dehydrogenase (G6PD) deficiency, which is common in most tropical areas. The recently registered tafenoquine is a slowly eliminated 8-aminoquinoline which does provide a single dose radical cure (although the currently recommended 300 mg adult dose appears to be too low). However, the price of the operational advantage of the single dose is the operational disadvantage of requiring a quantitative (rather than a qualitative) G6PD evaluation before treatment. This is because the slowly eliminated 8-aminoquinoline causes protracted haemolysis in G6PD deficiency, and this could be clinically significant even in female heterozygotes who may test as “normal” with the current rapid G6PD screens (i.e. the ‘spot” test or recently introduced rapid diagnostic tests) [9]. So tafenoquine is currently the only route to achieving the radical cure component of SERCAP on the near horizon [10].

Single dose treatment has to cure > 95% of non-immune patients reliably without incurring toxicity. For mass treatment (an original MalERA aspiration) it must be very safe and very well tolerated, as nearly all the recipients will be healthy and well. The current three-day artemisinin-based combination therapy (ACT) regimens are all spaced “loading doses” for the artemisinin partner drugs as they have to provide parasiticidal concentrations in blood for four asexual cycles (> 6 days) to ensure cure (Fig. 1). SERCAP has to do a lot better. It has to load safely and reliably with excellent tolerability in a single dose. The pharmacokinetic requirements are demanding: to reduce inter-individual variability in exposures, oral bioavailability for SERCAP drugs must be high, and ideally distribution volumes and clearance should vary little. Otherwise, high doses must be given to ensure adequate parasite killing in those patients with the lowest drug exposures. As for any anti-malarial drug, it is very important that efficacy (and tolerability) is established in patients with little or no immunity. In high transmission settings that means high cure rates must be obtained in young children. Efficacy should not be derived from studies in semi-immune older children and adults (who often self-cure and will have high cure rates with partially effective medicines), otherwise doses for non-immunes will be underestimated. Choosing the correct dosing regimen is critical, particularly for young children who often have relatively low exposures for a given mg/kg dose. We know from painful recent experience that anti-malarial drugs tend to be introduced at doses which are too low (mefloquine, artemether-lumefantrine, dihydroartemisin-piperaquine, tafenoquine). Elimination of the SERCAP drugs cannot be rapid either as minimum parasiticidal levels must be exceeded reliably for three (parasite reduction ratios > 10^4^) or four asexual cycles (parasite reduction ratios > 10^3^) i.e., 6 or 8 days respectively [11]. However, engineering slow elimination by increasing metabolic stability for drugs which generate bioactive reactive intermediates may result in reduced activity (e.g., artefenomel for arterolane, tafenoquine for primaquine). Overall SERCAP is a very “tough ask” in drug development, and so it may not be a wise target. New anti-malarial drugs are much needed, but it has not been easy to develop them [12]. With the notable exceptions of cipargamin and ganaplacide from the Novartis Institute for Tropical Diseases, most of the drugs in late clinical development (≥ phase 2) are old compounds discovered decades ago. The attrition rate is high, even after extensive preclinical development and, in some cases, after deployment (halofantrine, chlorproguanil-dapsone, artefenomel, DSM 265). It would be very unfortunate if a potential new anti-malarial drug was discarded early in development because it was eliminated rapidly, and so did not fit the SERCAP TPP.

**Fig. 1:**
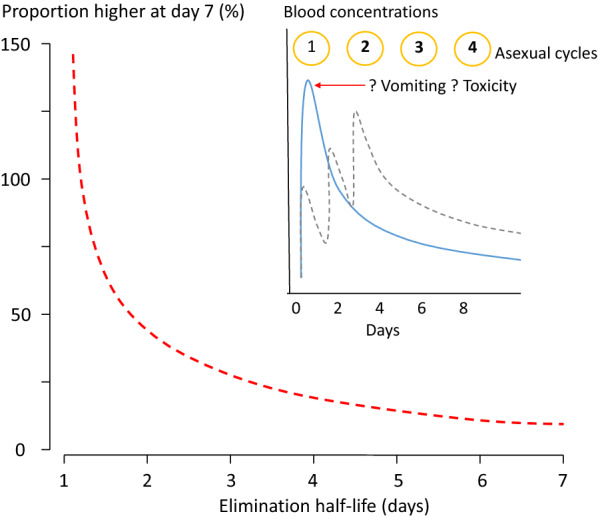
Proportion (%) by which anti-malarial drug blood concentrations are higher on day 7 (fourth post treatment asexual cycle) for a three-day regimen compared with a single dose in relation to the terminal elimination half-life (same total dose)

All the challenges described above are illustrated in the development of artefenomel-ferroquine by MMV and Sanofi. In 2007, after years of development, MMV decided to discontinue development of the first-generation synthetic peroxide arterolane (OZ 277) because it was considered to be too unstable. Development switched to a more stable and slowly eliminated peroxide compound (OZ 439; artefenomel). Since then, arterolane-piperaquine has been developed by Indian manufacturers into a successful 3-day ACT [13]. Initially piperaquine was also selected as the arterolane ACT partner, but there were concerns over cardiotoxicity risks with a large single dose and emerging piperaquine resistance in the greater Mekong subregion, so the piperaquine combination was not progressed. Ferroquine (a slowly eliminated modified 4-aminoquinoline with a novel ferrocene group providing good activity against chloroquine resistant *P. falciparum*), developed by Sanofi, was selected instead. The two were co-developed in the hope of providing a single encounter cure. Unfortunately, the extended phase 2B studies conducted between 2017 and 2019 were stopped early because of poor efficacy [14]. The single dose combination fared very poorly in Vietnam, where there is a high prevalence of artemisinin and piperaquine resistant *P. falciparum*, but it also failed to provide > 90% cure rates in African children. The poor efficacy in African children was driven by vomiting (mainly because of the artefenomel, which had to be administered in a large volume solution). Overall, one third of patients vomited and, unsurprisingly, these patients had low drug levels following the single dose administration (Fig. 2). Importantly, in those children who did achieve adequate drug levels, efficacy by Day 28 was excellent. What would have happened if this drug combination had been developed, not for the SERCAP TPP, but instead as a 3-day ACT?

**Fig. 2:**
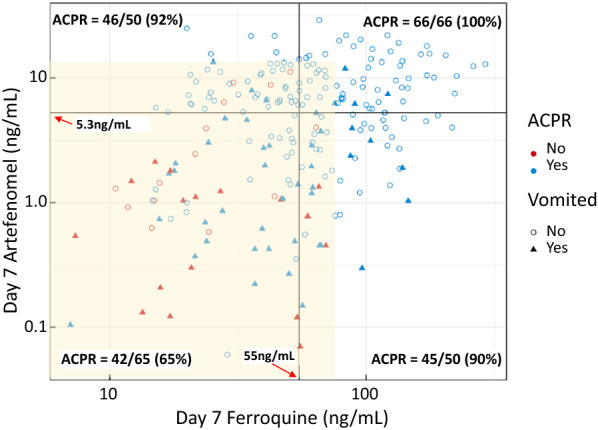
Estimated Day 7 concentrations of artefenomel and ferroquine measured in dry blood spots in African children ≤ 5y in relation to vomiting and PCR-adjusted ACPR at Day 28 in the prematurely discontinued extended phase 2 B studies [14]. The four quadrants were defined according to the median exposures. All patients with drug concentrations outside the yellow shaded area were “cured”

This is an unusual example having progressed so far. The influence of the SERCAP TPP is likely to have been greater at an earlier stage of drug discovery and development in lead optimization and candidate selection. There is nothing intrinsically wrong with aspiring to overcome a very difficult challenge (malaria elimination being a case in point). That is unless it prevents lesser, but still valuable, developments or improvements. We cannot say with certainty that the SERCAP TPP has been counterproductive, only that we suspect it may have been. We suggest that the TPP for new anti-malarial drugs is for a three-day regimen. If, in the future, drugs with suitable properties are discovered, and a 3-day regimen can be shortened safely without reducing efficacy, then that would be a welcome bonus.

## Data Availability

Not applicable.
